# Incidence of SARS‐CoV‐2 infection among COVID‐19 vaccinated and unvaccinated healthcare personnel, first responders, and other essential and frontline workers: Eight US locations, January–September 2021

**DOI:** 10.1111/irv.12956

**Published:** 2022-01-13

**Authors:** Allison L. Naleway, Lauren Grant, Alberto J. Caban‐Martinez, Meredith G. Wesley, Jefferey L. Burgess, Kimberly Groover, Manjusha Gaglani, Sarang K. Yoon, Harmony L. Tyner, Jennifer Meece, Jennifer L. Kuntz, Young M. Yoo, Natasha Schaefer‐Solle, Lauren E. W. Olsho, Joe K. Gerald, Spencer Rose, Matthew S. Thiese, Jessica Lundgren, Holly C. Groom, Josephine Mak, Paola Louzado Feliciano, Laura J. Edwards, Karen Lutrick, Kayan Dunnigan, Andrew L. Phillips, Julie Mayo Lamberte, Roger Noriega, Brian E. Sokol, Marilyn Odean, Katherine D. Ellingson, Michael Smith, Kurt T. Hegmann, Karley Respet, Monica Dickerson, Alexandra Cruz, Deanna E. Fleary, Kempapura Murthy, Angela Hunt, Eduardo Azziz‐Baumgartner, Damena Gallimore‐Wilson, Jenna A. Harder, Leah Odame‐Bamfo, Jennifer Viergutz, Melissa Arvay, John M. Jones, Peenaz Mistry, Mark G. Thompson, Ashley L. Fowlkes

**Affiliations:** ^1^ Kaiser Permanente Northwest Center for Health Research Portland Oregon USA; ^2^ Centers for Disease Control and Prevention COVID‐19 Response Team Atlanta Georgia USA; ^3^ Leonard M. Miller School of Medicine University of Miami Miami Florida USA; ^4^ Abt Associates, Inc Rockville Maryland USA; ^5^ Mel and Enid Zuckerman College of Public Health University of Arizona Tucson Arizona USA; ^6^ Baylor Scott and White Health Temple Texas USA; ^7^ Texas A&M University College of Medicine Temple Texas USA; ^8^ Rocky Mountain Center for Occupational and Environmental Health, Department of Family and Preventive Medicine University of Utah Health Salt Lake City Utah USA; ^9^ St. Luke's Regional Health Care System Duluth Minnesota USA; ^10^ Marshfield Clinic Research Institute Marshfield Wisconsin USA; ^11^ Whiteside Institute for Clinical Research, St. Luke's Duluth Minnesota USA

**Keywords:** essential worker, SARS‐CoV‐2, vaccine

## Abstract

**Background:**

We sought to evaluate the impact of changes in estimates of COVID‐19 vaccine effectiveness on the incidence of laboratory‐confirmed infection among frontline workers at high risk for SARS‐CoV‐2.

**Methods:**

We analyzed data from a prospective frontline worker cohort to estimate the incidence of COVID‐19 by month as well as the association of COVID‐19 vaccination, occupation, demographics, physical distancing, and mask use with infection risk. Participants completed baseline and quarterly surveys, and each week self‐collected mid‐turbinate nasal swabs and reported symptoms.

**Results:**

Among 1018 unvaccinated and 3531 fully vaccinated workers, the monthly incidence of laboratory‐confirmed SARS‐CoV‐2 infection in January 2021 was 13.9 (95% confidence interval [CI]: 10.4–17.4), declining to 0.5 (95% CI ‐0.4‐1.4) per 1000 person‐weeks in June. By September 2021, when the Delta variant predominated, incidence had once again risen to 13.6 (95% CI 7.8–19.4) per 1000 person‐weeks. In contrast, there was no reportable incidence among fully vaccinated participants at the end of January 2021, and incidence remained low until September 2021 when it rose modestly to 4.1 (95% CI 1.9–3.8) per 1000. Below average facemask use was associated with a higher risk of infection for unvaccinated participants during exposure to persons who may have COVID‐19 and vaccinated participants during hours in the community.

**Conclusions:**

COVID‐19 vaccination was significantly associated with a lower risk of SARS‐CoV‐2 infection despite Delta variant predominance. Our data demonstrate the added protective benefit of facemask use among both unvaccinated and vaccinated frontline workers.

## 1 BACKGROUND

COVID‐19 vaccines have shown >90% effectiveness in preventing SARS‐CoV‐2 infection, but estimates of vaccine effectiveness (VE) declined to 66% during B.1.617.2 (Delta) predominance.^1^, ^2^, ^3^, ^4^, ^5^ Changes in COVID‐19 VE estimates associated with changes in predominating variant virus circulation over time may have important implications for infection risk in work and community settings. Particularly at increased risk of SARS‐CoV‐2 infection are frontline workers, including healthcare personnel, first responders, and essential and other frontline workers who provide direct care and services to the public. Using data from the CDC HEROES‐RECOVER Network, a prospective frontline worker cohort, we examined changes in the monthly incidence of COVID‐19 by vaccination status, as well as the association of additional factors of occupation, demographics, physical distancing, and mask use with risk of infection. Here, we demonstrate that COVID‐19 vaccines dramatically reduced the risk of any SARS‐CoV‐2 infection among vaccinated adults at high occupational risk of exposure, and substantial protection continues to be evident despite the high prevalence of the Delta variant during recent months.

## 2 METHODS

The HEROES‐RECOVER Network includes Arizona Healthcare, Emergency Response, and Other Essential Workers Surveillance Study (HEROES) conducted in Phoenix, Tucson, and other noncentrally located areas in Arizona and Research on the Epidemiology of SARS‐CoV‐2 in Essential Response Personnel (RECOVER) conducted in Miami, Florida, Duluth, Minnesota, Portland, Oregon, Temple, Texas, and Salt Lake City, Utah. Active surveillance is conducted with weekly participant or medical record reports for COVID‐19‐like illness (CLI) defined as ≥1 symptom of fever, chills, cough, shortness of breath, sore throat, diarrhea, muscle aches, or change in smell or taste.^6^, ^7^ As rotating questions in weekly surveys, participants reported hours in the past 7 days of direct contact (within 3 ft.) and associated percentage facemask use with people at work, in the community, or in any setting who may have COVID‐19. Hours of direct contact and percent facemask use were summarized as monthly averages, and then each week was categorized as above or below the corresponding monthly average. Participants self‐collected a mid‐turbinate nasal swab each week and at the onset of CLI for SARS‐CoV‐2 testing by reverse transcription–polymerase chain reaction (RT‐PCR) and genomic sequencing, if positive. COVID‐19 vaccinations were documented through vaccine cards, electronic medical records, or state immunization registries. Participants were considered fully vaccinated ≥14 days after a second mRNA COVID‐19 vaccine dose or a single Johnson & Johnson's Janssen vaccine dose; unvaccinated participants had no COVID‐19 vaccine receipt. Indeterminate vaccination status was defined from one day following a COVID‐19 vaccine dose until the participant was fully vaccinated and was excluded from analysis. Participants provided informed consent for study activities, and the study protocol was reviewed and approved by institutional review boards at all participating sites, including Abt Associates and CDC.

From January to September 2021, participants with no prior SARS‐CoV‐2 infection contributed unvaccinated or fully vaccinated person‐time according to standard definitions until SARS‐CoV‐2 detection, study withdrawal, or the end of the study period. Monthly unadjusted incidence was calculated as the number of infections relative to the observed person‐weeks, and 95% Wald confidence intervals were estimated. To examine factors associated with SARS‐CoV‐2 incidence during the entire observation period stratified by vaccination status, we used stratified Poisson generalized estimating equations to account for repeated measures.^8^ Each model had the factor of interest as the independent variable, log weeks of exposure as an offset, and participants repeating with an independent correlation structure. Statistical significance was defined as a two‐tailed *p*‐value < 0.05. A final model examined incidence by vaccination status, adjusted for factors significantly associated with infection in univariate analysis, including site, occupation, and reported direct contact and facemask use. All analyses were conducted using SAS Version 9.4 and graphics created using R Version 4.1.

## 3 RESULTS

Among 4549 participants, 1020 (22%) were unvaccinated, and 3529 (78%) were fully vaccinated by the end of the observation period (Table 1); 2302 (65%) received Pfizer‐BioNTech mRNA, 1144 (32%) Moderna mRNA‐1273, and 83 (2%) J&J/Janssen COVID‐19 vaccines. Overall, 48% of participants were located in Arizona study sites, 62% female, 73% aged 18–49 years, 95% white, 18% Hispanic, and 25% had at least one chronic medical condition. Approximately half of participants were healthcare workers, 20% were first responders, and 28% were other frontline workers. Participants reported a monthly average of 9 direct contact hours and 97% facemask use with persons known or suspected to have COVID‐19; facemask use was lower during contact with coworkers, patients, or the public at work (88%) or in the community (76%) when COVID‐19 was not suspected (data not shown). The frequency in which a participant's report was higher or lower than the average is given in Table 1.

**TABLE 1:** Characteristics of health care personnel, first responders, and other essential and frontline workers with reverse transcription–polymerase chain reaction (RT‐PCR)‐confirmed SARS‐CoV‐2 infections and percentage receiving one or more doses of COVID vaccine—January–September 2021

						COVID‐19 vaccination status				
Characteristics	Total participants		SARS‐CoV‐2 infections			Unvaccinated		Fully vaccinated^a^		
	No.	Distribution (%)	No.	(%)	*P*‐value	No.	(%)	No.	(%)	*P*‐value
All participants	4549		257	(5.6)		1018	(22.4)	3531	(77.6)	
Sociodemographic characteristics										
Cohort location										
Phoenix, AZ	519	(11.4)	42	(8.1)	<0.0001	139	(26.8)	380	(73.2)	<0.0001
Tucson, AZ	1320	(29.0)	52	(3.9)		378	(28.6)	942	(71.4)	
Other areas in AZ	330	(7.3)	14	(4.2)		109	(33.0)	221	(67.0)	
Miami, FL	407	(8.9)	44	(10.8)		162	(39.8)	245	(60.2)	
Duluth, MN	544	(12.0)	18	(3.3)		30	(5.5)	514	(94.5)	
Portland, OR	551	(12.1)	17	(3.1)		40	(7.3)	511	(92.7)	
Temple, TX	329	(7.2)	31	(9.4)		59	(17.9)	270	(82.1)	
Salt Lake City, UT	549	(12.1)	39	(7.1)		101	(18.4)	448	(81.6)	
Sex^b^										
Female	2810	(61.8)	139	(4.9)	0.0090	572	(20.4)	2238	(79.6)	<0.0001
Male	1739	(38.2)	118	(6.8)		446	(25.6)	1293	(74.4)	
Age (years)										
18–49	3301	(72.6)	193	(5.8)	0.3490	803	(24.3)	2498	(75.7)	<0.0001
≥50	1248	(27.4)	64	(5.1)		215	(17.2)	1033	(82.8)	
Race										
White	4295	(94.4)	249	(5.8)	0.0757	926	(21.6)	3369	(78.4)	<0.0001
Other	254	(5.6)	8	(3.1)		92	(36.2)	162	(63.8)	
Ethnicity										
Hispanic/Latino	817	(18.0)	54	(6.6)	0.1895	282	(34.5)	535	(65.5)	<0.0001
Other	3732	(82.0)	203	(5.4)		736	(19.7)	2996	(80.3)	
Occupation^c^										
Primary HCP	868	(19.1)	32	(3.7)	<0.0001	56	(6.5)	812	(93.5)	<0.0001
Nurses and other allied HCP	1450	(31.9)	69	(4.8)		251	(17.3)	1199	(82.7)	
First responders	953	(20.9)	92	(9.7)		330	(34.6)	623	(65.4)	
Essential and other frontline	1278	(28.1)	64	(5.0)		381	(29.8)	897	(70.2)	
Chronic condition^d^										
None	3427	(75.3)	190	(5.5)	0.5905	808	(23.6)	2619	(76.4)	0.0008
1 or more	1122	(24.7)	67	(6.0)		210	(18.7)	912	(81.3)	
Direct contact and facemask use^e^										
Contact with COVID‐19										
Hours (*n* = 4477)^f^										
Above monthly mean	1337	(29.8)	99	(7.4)	0.0009	345	(25.8)	992	(74.2)	<0.0001
Below monthly mean	3140	(70.1)	154	(4.9)		624	(19.9)	2516	(80.1)	
Percent facemask use (*n* = 4486)										
Above monthly mean	3740	(83.4)	192	(5.1)	0.0010	746	(19.9)	2994	(80.1)	<0.0001
Below monthly mean	746	(16.6)	61	(8.2)		228	(30.6)	518	(69.4)	
Contact with people at work										
Hours (n = 4509)										
Above monthly mean	2194	(48.7)	133	(6.1)	0.2498	500	(22.8)	1694	(77.2)	0.1352
Below monthly mean	2315	(51.3)	122	(5.3)		485	(21.0)	1830	(79.0)	
Percent facemask use										
Above monthly mean	3139	(70.6)	141	(4.5)	<0.0001	550	(17.5)	2589	(82.5)	<0.0001
Below monthly mean	1308	(29.4)	111	(8.5)		400	(30.6)	908	(69.4)	
Contact with people in the community										
Hours (*n* = 4304)										
Above monthly mean	1844	(42.8)	115	(6.2)	0.2476	456	(24.7)	1388	(75.3)	<0.0001
Below monthly mean	2460	(57.2)	133	(5.4)		428	(17.4)	2032	(82.6)	
Percent mask use (*n* = 4506)										
Above monthly mean	2669	(59.2)	119	(4.5)	<0.0001	516	(19.3)	2153	(80.7)	<0.0001
Below monthly mean	1837	(40.7)	137	(7.5)		471	(25.6)	1366	(74.4)	

Abbreviation: HCP, healthcare personnel.

^a^ Participants were considered fully vaccinated 14 days after receipt of a second mRNA COVID‐19 vaccine dose (2302 [65%] Pfizer‐BioNTech and 1144 [32%] Moderna recipients), or a single Johnson & Johnson's Janssen vaccine dose (83 [2%] recipients). Vaccination status shown represents participant status at the end of the observation period.

^b^ For 15 participants missing biological sex, it was imputed as the more common category (female).

^c^ Occupation categories: primary HCP (physicians, physician assistants, nurse practitioners, dentists), other allied HCP (nurses, therapists, technicians, medical assistants, orderlies, and all others providing clinical support in inpatient or outpatient settings), first responders (firefighters, law enforcement, corrections, emergency medical technicians), essential and other frontline workers (workers in hospitality, delivery, and retail; teachers; all other occupations that require contact within 3 ft. of the public, customers, or coworkers as a routine part of their job).

^d^ Chronic conditions included asthma; other chronic lung disease, such as COPD, emphysema; cancer; diabetes; heart disease or condition; hypertension or high blood pressure; immunosuppression; kidney disease; liver disease; neurologic or neuromuscular disease or disorder; and autoimmune disease. For 135 participants, who did not respond to the self‐report question, they were imputed as none, pending further verification.

^e^ Each week, participants were asked to report hours of direct contact (within 3 ft.) of others and percent of these hours wearing a facemask in the past 7 days in one of the following scenarios: with persons who have confirmed or suspected COVID‐19, people at work, or people in the community.

^f^ For individual questions, denominators vary due to missing information: Participants may have withdrawn prior to answering, newer enrollees may not have received the question at the time of this analysis, or participants may choose not to answer.

Frequency of SARS‐CoV‐2 infections varied by site and was significantly higher among participants who were male, first responders, and reported lower than average facemask use (Table 1). Receipt of COVID‐19 vaccination also varied by site and was significantly higher among participants who were female, aged ≥50 years, white, non‐Hispanic, healthcare personnel, had at least one chronic condition, and reported fewer hours of direct contact and greater use of facemasks with others at work and in the community (Table 1).

**FIGURE 1:**
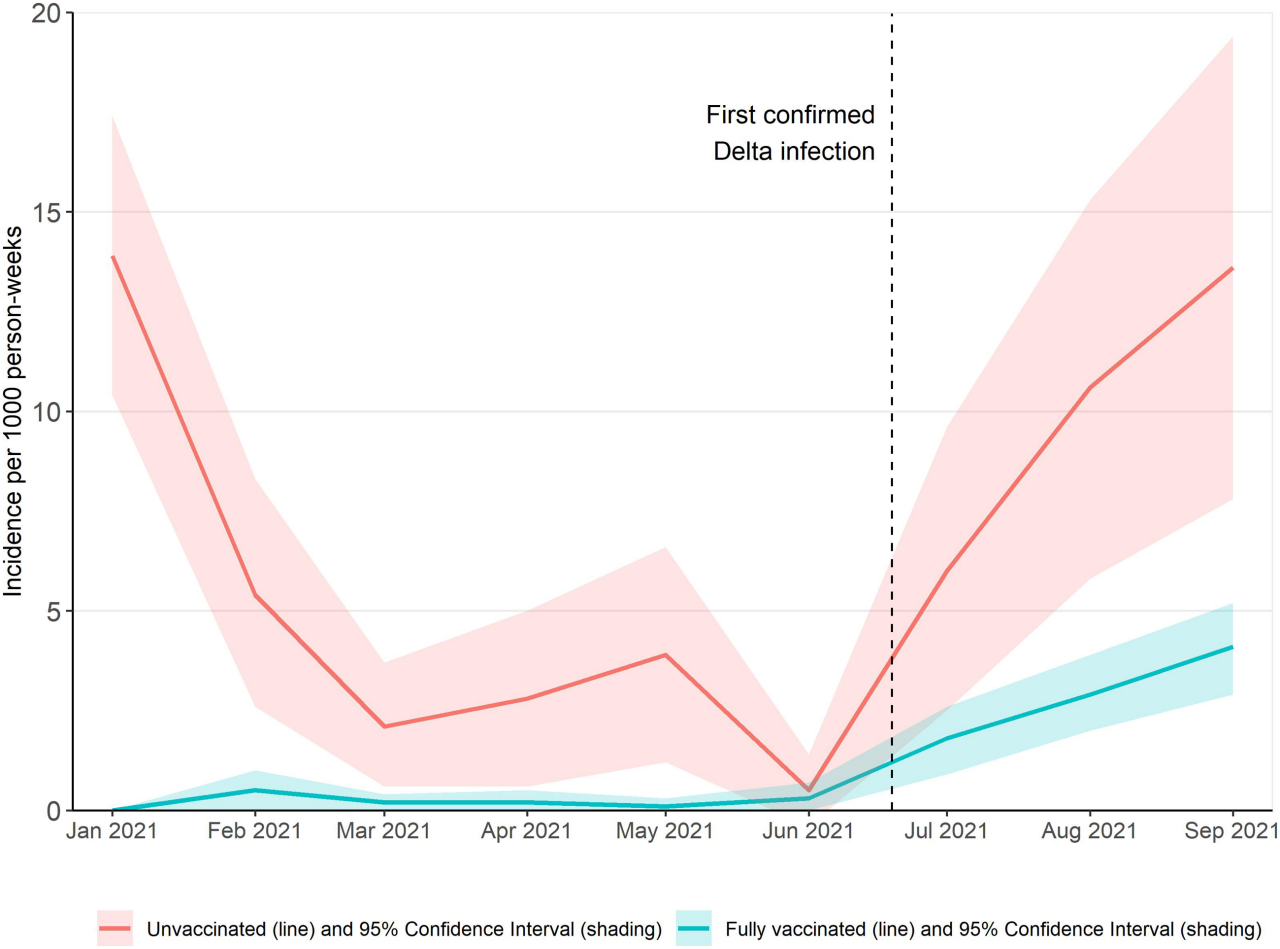
Monthly incidence^*^ of SARS‐CoV‐2 among unvaccinated and fully COVID‐19 vaccinated frontline workers—January–September 2021. The unadjusted incidence was calculated for each month as the number of infections relative to the observed person‐weeks, and 95% confidence intervals were estimated using Wald intervals

Monthly incidence rates with 95% confidence intervals were plotted for unvaccinated and vaccinated adults during January to September 2021 (Figure 1). Among unvaccinated adults, incidence rates had a W‐shaped trend with the highest incidence of SARS‐CoV‐2 infection in both January (13.9 per 1000 person‐weeks, 95% CI 10.4–17.4) and September (during Delta variant predominance) (13.6 per 1000 person‐weeks, 95% CI: 7.8–19.4) and a smaller peak in May (3.9 per 1000 person‐weeks, 95% CI 1.2–6.6). In contrast, incidence of SARS‐CoV‐2 infection among fully vaccinated adults remained very low throughout the study period until the Delta variant became predominant with peak incidence in September (4.1 per 1000 person‐weeks, 95% CI 1.9–3.8).

First responders had the highest risk of infection, regardless of vaccination status (Table 2). The increased risk of SARS‐CoV‐2 infection among unvaccinated and vaccinated adults was greatest in Phoenix, Arizona, and Florida; among vaccinated participants, rates were also higher in Texas and Utah. Among vaccinated and unvaccinated participants, infection risk was also increased when reporting above average hours of contact with persons who had confirmed or suspected COVID‐19 or below average facemask use during contact. Vaccinated participants similarly had an increased risk when reporting above average hours of COVID‐19 contact or below average facemask use during direct contact with people at work or in the community; these patterns were similar for unvaccinated participants but did not reach statistical significance.

**TABLE 2:** Incidence^a^ of SARS‐CoV‐2 infection among unvaccinated and fully COVID‐19 vaccinated frontline workers by sociodemographic and behavioral factors—January–September 2021

	COVID‐19 vaccination status			
Characteristic	Unvaccinated		Fully vaccinated	
	Cumulative incidence*	Relative rate (95% CI)	Cumulative incidence*	Relative rate (95% CI)
Sociodemographics				
Cohort location				
Tucson, AZ	5.2	(Referent)	0.7	(Referent)
Phoenix, AZ	**9.7**	**1.8 (1.1–3.0)**	**1.9**	**2.6 (1.3–5.0)**
Other areas in AZ	4.9	0.9 (0.4–1.7)	0.8	1 (0.4–3.1)
Miami, FL	**12.4**	**2.3 (1.4–3.7)**	**3.1**	**4.3 (2.1–8.9)**
Duluth, MN	3.6	0.7 (0.2–1.9)	1.1	1.5 (0.7–3.1)
Portland, OR	4.2	0.8 (0.3–1.8)	0.8	1.1 (0.5–2.4)
Temple, TX	8.3	1.5 (0.8–2.9)	**2.9**	**4 (2.1–7.8)**
Salt Lake City, UT	6.0	1.1 (0.7–1.9)	**1.6**	**2.2 (1.1–4.3)**
Occupation^b^				
Primary HCP	4.7	1 (0.4–2.2)	1.1	0.9 (0.5–1.5)
Nurses and other allied HCP	5.9	1.2 (0.8–1.9)	1.1	0.8 (0.5–1.4)
First responders	10.6	2.2 (1.5–3.2)	2.1	1.6 (1.0–2.8)
Essential and other frontline	4.9	(Referent)	1.3	(Referent)
Sex				
Female	6.1	0.8 (0.6–1.1)	1.2	0.7 (0.5–1.1)
Male	7.5	(Referent)	1.6	(Referent)
Age (years)				
18–49	7.1	1.2 (0.8–1.8)	1.4	1.1 (0.8–1.7)
≥50	5.7	(Referent)	1.2	(Referent)
Race				
White	6.8	1.3 (0.6–2.7)	0.9	‐‐
Other	6.0	(Referent)	0	(Referent)
Ethnicity				
Hispanic/Latinx	7.7	1.2 (0.8–1.7)	1.3	1 (0.6–1.7)
Other	6.4	(Referent)	1.3	(Referent)
Chronic condition				
None	6.6	0.9 (0.6–1.3)	1.3	1 (0.6–1.4)
1 or more	7.2	(Referent)	1.3	(Referent)
Direct contact and facemask use^c^				
Contact with COVID‐19				
Hours				
Above monthly mean	9.8	**1.8 (1.3–2.6)**	2.6	2.7 (1.8–3.9)
Below monthly mean	5.3	(Referent)	0.9	(Referent)
Percent facemask use				
Above monthly mean	**6.1**	(Referent)	1.3	(Referent)
Below monthly mean	**12.7**	**2.1 (1.3–3.3)**	2.2	1.7 (0.8–3.9)
Contact with people at work				
Hours				
Above monthly mean	5.9	0.8 (0.5–1.1)	1.1	0.7 (0.5–1.0)
Below monthly mean	7.8	(Referent)	1.6	(Referent)
Percent facemask use				
Above monthly mean	6.0	(Referent)	**1.2**	(Referent)
Below monthly mean	8.0	1.3 (1.0–1.9)	**2.0**	**1.7 (1.1–2.7)**
Contact with people in the community				
Hours				
Above monthly mean	6.5	1 (0.7–1.3)	1.2	1 (0.7–1.4)
Below monthly mean	6.7	(Referent)	1.3	(Referent)
Percent facemask use				
Above monthly mean	6.2	(Referent)	**1.1**	(Referent)
Below monthly mean	7.8	1.3 (0.9–1.8)	**1.9**	**1.9 (1.3–2.7)**

Abbreviations: AZ, Arizona; CI, confidence interval; COVID‐19, coronavirus disease 2019; FL, Florida; HCP, healthcare personnel; MN, Minnesota; OR, Oregon; TX, Texas; UT, Utah.

^a^ Unadjusted cumulative incidence was calculated as the number of cases relative to the observed person‐weeks using stratified Poisson generalized estimating equations to account for repeated measures.

^b^ Occupation categories: primary HCP (physicians, physician assistants, nurse practitioners, dentists), other allied HCP (nurses, therapists, technicians, medical assistants, orderlies, and all others providing clinical support in inpatient or outpatient settings), first responders (firefighters, law enforcement, corrections, emergency medical technicians), essential and other frontline workers (workers in hospitality, delivery, and retail; teachers; all other occupations that require contact within 3 ft. of the public, customers or coworkers as a routine part of their job).

^c^ Each week, participants were asked to report hours of direct contact (within 3 ft.) of others and percent of these hours wearing a facemask in the past 7 days in one of the following scenarios: with persons who have confirmed or suspected COVID‐19, people at work, or people in the community.

The cumulative adjusted incidence of SARS‐CoV‐2 infection among unvaccinated participants was 7.6 (95% CI 6.4–9.1) compared with 1.9 (95% CI 1.5–2.3) among vaccinated participants, indicating the relative risk of SARS‐CoV‐2 infection among unvaccinated participants 4.1 (95% CI 3.1–5.3) times higher than fully vaccinated participants.

## 4 DISCUSSION

In a prospective cohort of frontline workers, the risk of SARS‐CoV‐2 infection, regardless of symptoms, was four times higher for unvaccinated adults (7.6 per 1000 person‐weeks) compared with vaccinated (1.9 per 1000 person‐weeks). Although the rate of infection among vaccinated participants remained below 1 per 1000 person‐weeks for the first half of 2021, we observed a concomitant increase in rates with increasing Delta variant prevalence that was pronounced among unvaccinated and modest among vaccinated participants. These findings of continued lower risk of infection among the fully vaccinated relative to the unvaccinated, including during weeks when the Delta variant was predominant, are consistent with previously reported highly protective estimates of COVID‐19 VE against infection.^2^, ^9^, ^10^

A strength of the current analysis is the inclusion of multiple measures of direct contact with known or suspected COVID‐19 cases, coworkers, and the public, as well as reported facemask (or other protective equipment) use in these settings. The gap in risk of SARS‐CoV‐2 infection between unvaccinated and vaccinated adults was heightened by the additional protective benefit of facemask use. Unvaccinated participants reported lower facemask use during hours of direct contact with COVID‐19 or with people at work or in the community, consistent with reported lower overall adherence with infection control measures among unvaccinated persons.^11^ These findings underscore the importance of interpreting differences in incidence estimates by vaccination status in the context of individual physical distancing and facemask use behaviors in addition to observed differences by occupation and site location.

The findings in this study are subject to four key limitations. First, the HEROES‐RECOVER participants represent a highly vaccinated population relative to the general population, and the person‐time contribution of unvaccinated participants is diminished and may not indicate statistical significance for all analyses. Second, if COVID‐19 vaccination reduces nasal viral RNA load,^5^ the sensitivity of virus detection by RT‐PCR could be reduced and underestimate incidence in the vaccinated. Third, self‐collection of specimens or shipping delays, though rare, could compromise specimen quality and further lead to reduced RT‐PCR sensitivity and underestimation of incidence. Fourth, the prospective study design is reliant upon participant adherence to weekly specimen collection and symptom reporting that is rarely perfect. However, HEROES‐RECOVER has maintained a very high level of adherence to weekly symptom reporting and swab collection (median 100%; interquartile range 82%–100%).

HEROES‐RECOVER network completes routine weekly testing for SARS‐CoV‐2 regardless of symptoms and collects detailed information from participants, allowing for estimation of incidence with laboratory‐confirmed outcomes accounting for the impact of individual characteristics and behaviors. The high effectiveness of COVID‐19 vaccines (noted in clinical trials and in real‐world estimates from this network) translate into substantial decreases in the actual number of adults who are infected following vaccination. Our findings further support the benefit of full vaccination and ongoing interventions to improve vaccine uptake and adherence to infection control measures, such as physical distancing and masking.

## AUTHOR CONTRIBUTIONS

**Allison Naleway:** Conceptualization; data curation; investigation; methodology. **Lauren Grant:** Formal analysis; investigation; methodology. **Alberto Caban‐Martinez:** Data curation; investigation. **Meredith Wesley:** Data curation; formal analysis; investigation. **Jefferey Burgess:** Conceptualization; data curation; investigation. **Kimberly Groover:** Data curation; investigation. **Manjusha Gaglani:** Conceptualization; data curation; investigation. **Sarang Yoon:** Conceptualization; data curation; investigation. **Harmony Tyner:** Conceptualization; data curation; investigation. **Jennifer Meece:** Data curation; investigation. **Jennifer Kuntz:** Data curation; investigation. **Young Yoo:** Formal analysis; investigation; methodology. **Natasha Schaefer‐Solle:** Data curation; investigation. **Lauren Olsho:** Conceptualization; investigation; methodology. **Joe Gerald:** Data curation; investigation. **Spencer Rose:** Data curation; investigation. **Matthew Thiese:** Data curation; investigation. **Jessica Lundgren:** Data curation; investigation. **Holly Groom:** Data curation; investigation. **Josephine Mak:** Formal analysis; investigation. **Paola Feliciano:** Data curation; investigation. **Laura Edwards:** Data curation; investigation. **Karen Lutrick:** Data curation; investigation. **Kayan Dunnigan:** Data curation; investigation. **Andrew Phillips:** Data curation; investigation. **Marilyn Odean:** Data curation; investigation. **Katherine Ellingson:** Data curation; investigation. **Karley Respet:** Data curation; investigation. **Mark Thompson:** Conceptualization; investigation; methodology. **Ashley Fowlkes:** Conceptualization; investigation; methodology.

## CONFLICT OF INTEREST

All authors have completed and submitted the International Committee of Medical Journal Editors form for disclosure of potential conflicts of interest. Allison L. Naleway reported institutional funding from Pfizer and Vir Biotechnology for studies unrelated to the submitted work and Jennifer Kuntz receives research funding from Pfizer, Novartis, and Vir Biotechnology for unrelated studies. Matthew S. Thiese reports grants and personal fees from Reed Group and the American College of Occupational and Environmental Medicine, outside the submitted work. No other potential conflicts of interest were disclosed. The findings and conclusions in this report are those of the authors and do not necessarily represent the official position of the Centers for Disease Control and Prevention.

### PEER REVIEW

The peer review history for this article is available at https://publons.com/publon/10.1111/irv.12956.

## Data Availability

The data that support the findings of this study are available from the corresponding author upon reasonable request.
